# Chest CT Findings and Their Temporal Evolution in COVID-19 Pneumonia

**DOI:** 10.7759/cureus.26021

**Published:** 2022-06-16

**Authors:** Sandhya K Hemraj, M. J Jacob, Vidyashree Kotian, Sachin D K., Geetha R G., Lilly B Veliath

**Affiliations:** 1 Department of Radiology, MVJ (M.V. Jayaraman) Medical College and Research Hospital, Bangalore, IND; 2 Department of General Medicine, MVJ (M.V. Jayaraman) Medical College and Research Hospital, Bangalore, IND

**Keywords:** covid-19, covid-19 pandemic, temporal evolution, consolidation, ground glass opacities, ct chest

## Abstract

Introduction: The coronavirus disease 2019 (COVID-19) pandemic originated in China in November 2019 and is caused by the SARS-CoV-2 virus. The virus binds to nasal and pharyngeal epithelial cells and migrates to the lower respiratory tract. The confirmatory test for COVID-19 infection is the reverse transcription-polymerase chain reaction (RT-PCR). Chest CT plays an important role in the diagnosis, triage, and treatment of affected individuals. We describe the findings on chest CT and their temporal evolution in COVID-19 pneumonia.

Methods: We conducted a retrospective, cross-sectional study on COVID-19-positive patients who underwent chest CT. CT images of the patients were reviewed for ground-glass opacities, consolidation, crazy-paving appearance, vascular dilatation, traction bronchiectasis, architectural distortion, and subpleural and parenchymal bands. Distribution of opacities on axial sections, ancillary findings, and co-existent lung diseases were recorded. To assess the temporal evolution of CT findings, the time in days between the onset of the first symptom and the date of the CT scan of each patient was recorded. Statistical analysis was performed.

Results: Ground-glass opacities, consolidation, and a combination of both were the most important features in COVID-19 pneumonia. Patients in the early stage showed simple ground-glass opacities; in the progressive stage showed consolidation and ground-glass opacities with crazy-paving appearance, subpleural and parenchymal bands, and architectural distortion; in the peak stage showed progression of these findings; and in the late stage showed interval resolution of these findings. Axial distribution of these opacities was asymmetric, with peripheral subpleural predominance involving posterior, lateral, and both these locations, associated with apicobasal gradient.

Conclusion: Chest CT permits rapid diagnosis of COVID-19 pneumonia, enabling appropriate treatment to be instituted at the earliest. Thus, it is life-saving in resource-constrained environments.

## Introduction

The coronavirus disease 2019 (COVID-19) pandemic has taken the world by storm and had an unprecedented, devastating effect on the human population globally. The infection likely originated from Wuhan, the capital city of Hubei province in central China in late November 2019 [1]. It is caused by the severe acute respiratory syndrome coronavirus 2 (SARS-CoV-2). To date, there have been about 5,330,000 deaths worldwide and about 476,000 deaths in India directly attributable to this viral infection. The infectious outbreak was initially declared a “public health emergency of international concern” by the WHO on January 30, 2020, and subsequently a pandemic on March 11, 2020 [2].

This is the seventh coronavirus to infect human beings [3]. Two other coronavirus infections include the severe acute respiratory syndrome and the Middle East respiratory syndrome, both of which have zoonotic origins; the former began in southern China and the latter originated in Saudi Arabia [3]. Person-to-person transmission of the SARS-CoV-2 virus is well established through direct and indirect air-borne droplet infection of respiratory secretions [4].

In an infected individual, the inhaled virus particles bind to the ciliated secretory cells in the nasopharyngeal epithelium via the angiotensin-converting enzyme 2 (ACE2) receptors. Here, they replicate, locally propagate, and then migrate to the lower respiratory tract. The immune response mounted by an individual’s body is sufficient to contain the further spread of infection in about 80% of infected individuals. The initial infection is confined to the upper respiratory tract, with viral clearance in 10 to 14 days. In the remaining 20%, the virus migrates to the lower respiratory tract via conducting airways and infects the type 2 alveolar epithelial cells. This is when the patient starts developing symptoms. The virus-laden pneumocytes release cytokines, interleukins, tumor necrosis factors, interferons, and several pro-inflammatory proteins [5]. This “cytokine storm” acts as a chemo-attractant for neutrophils, CD4, and CD8 cells, which get sequestered in the lungs. These inflammatory cells attempt to ward off the infection, concurrently inducing pulmonary parenchymal inflammation and injury [5]. The vicious cycle of host cell apoptosis, the release of new viral particles, and infection of adjacent type 2 alveolar epithelial cells if left uncontrolled becomes self-propagating. Persistent inflammatory cell injury and viral replication lead to loss of pulmonary alveolar pneumocytes and diffuse alveolar damage, eventually culminating in acute respiratory distress syndrome (ARDS) [6,7].

The symptoms of COVID-19 infection include fever, cough, fatigue, myalgia, shortness of breath, and sore throat [8]. The majority of infected patients remain asymptomatic [8]. In a small subset of infected individuals, the infection may run a severe course, characterized by dyspnoea and/or hypoxemia that quickly progresses to ARDS, septic shock, metabolic acidosis, and coagulation disorders. These complications require hospital admission and intensive care monitoring, and can eventually lead to death in about 4-15% of all infected patients.

The “gold standard” test for the diagnosis of COVID-19 infection is the reverse transcription-polymerase chain reaction (RT-PCR) performed on specimens obtained from the upper respiratory tract or blood during the first week of symptoms [9]. Other commonly encountered laboratory findings include lymphopenia, eosinopenia, altered neutrophil/lymphocyte ratio of ≥ 3.13, thrombocytopenia, elevated levels of C-reactive protein (CRP), ferritin, D-dimer, procalcitonin, prothrombin time (PT), activated partial thromboplastin time (aPTT), creatine kinase, serum glutamic pyruvic transaminase (SGPT), serum urea, and creatinine. These biochemical and systemic abnormalities indicate a propensity to develop thromboembolic complications and myocardial, hepatic, and renal damage [9].

A clear understanding of clinical presentation, diagnosis, classification of disease severity, and appropriate management of confirmed cases is of vital importance in the control of the infectious outbreak. Several research studies have pointed out the role of chest CT scans in early diagnosis, triage, and institution of prompt, appropriate treatment and containment measures in affected individuals. The present research study attempts to describe the findings on chest CT and their temporal evolution in COVID-19 pneumonia.

This study aims to describe the findings on chest CT scans in COVID-19 pneumonia and to determine the sequential temporal changes in CT scan findings in relation to the time duration of COVID-19 pneumonia.

## Materials and methods

Study design

This was a retrospective, cross-sectional, descriptive study. The study population consisted of 103 adult patients who presented to the hospital with clinical features suggestive of severe acute respiratory infection and met the prevalent WHO clinical criteria for case definition of acute COVID-19 infection. They also tested positive with the RT-PCR test for COVID-19 infection and underwent chest CT scans from July 2020 to April 2021.

Techniques and data collection

A 16-slice multidetector computed tomography (MDCT) scanner (GE Brivo CT385, GE Healthcare, Chicago, IL) was used for performing the chest CT scans. Patients were scanned in the supine position in the craniocaudal direction and breath-hold images at end inspiration were obtained. Where patients were breathless or could not hold their breath for a sufficiently long time, segmented CT scan images were acquired. Children and severely breathless adults who could not undergo the CT scan were excluded from the study. After the acquisition, the CT images were reconstructed retrospectively in high-resolution computed tomography (HRCT) kernel using a high spatial frequency algorithm, with 1.25 mm slice thickness at 1.25 mm intervals, covering the entire thorax from the thoracic inlet superiorly to just below domes of diaphragm inferiorly. Scanning parameters were X-ray tube current at 120 to 140 kVp, 80-100 mA. The images were reconstructed in lung and mediastinal window settings. Proper disinfection protocol was followed after the completion of chest CT scans at the end of each day. The CT images were reviewed and analyzed and findings were documented as per the structured tabulated study proforma. The time in days between symptom onset and the date of CT scan of each patient was recorded.

This study was approved by the Institutional Scientific and Ethical Review Committees of MVJ Medical College and Research Hospital vide letter number MVJMC&RH/PG/Synopsis/5/2021-22, dated July 30, 2021. Written informed consent in English and vernacular language was taken from all patients prior to initiation of the CT scans. All the data collected in this study including patients’ names and relevant clinical and imaging records were maintained strictly confidential.

Review of CT scan images

Four radiologists conducted a preliminary independent review of all the patients’ chest CT images and the final review of images was conducted by a single radiologist in consensus with the findings in the preliminary review. The chest CT scan images were analyzed for the presence or absence of ground-glass opacities, consolidation, crazy-paving appearance, vascular dilatation, traction bronchiectasis, architectural distortion, and subpleural and parenchymal bands. Ancillary findings like pleural thickening/effusion/pericardial effusion, mediastinal lymphadenopathy, cavitation, and CT halo sign, if any, were also recorded. Co-existence of underlying lung diseases, such as emphysema, chronic obstructive pulmonary disease, and pulmonary tuberculosis, if present, were also documented. The distribution of opacities on axial sections including unilateral or bilateral, anterior or posterior predominance, central, peripheral, or diffuse distribution was also recorded in each patient.

To assess the temporal evolution of CT findings, the time in days between the onset of first symptom and date of CT scan of each patient in symptomatic patients or date of positive RT-PCR report in asymptomatic patients were recorded from the case sheets. Based on this, all the study patients were classified into four groups: early, progressive, peak, and late stages. Patients in whom chest CT was performed between one and four days after symptom onset belonged to the early stage, between five and eight days after symptom onset belonged to the progressive stage, between nine and 13 days after symptom onset belonged to the peak stage, and on or after 14 days following symptom onset belonged to the late stage [10].

Statistical analysis

Statistical analysis was performed using the IBM SPSS package version 22.0 (IBM Corp., Armonk, NY). The variables collected and analyzed were patient demographics, salient CT findings described above, ancillary findings, and underlying lung diseases. The prevalence of ground-glass opacities and consolidation was correlated with the total number of patients in each stage of COVID-19 pneumonia. Each CT finding was also correlated with the time duration of the chest CT from the date of symptom onset. The distribution of opacities whether unilateral or bilateral, anterior or posterior predominance, central, peripheral, or diffuse distribution in the lung was also analyzed. The descriptive statistics for quantitative data are expressed as mean and standard deviation and qualitative data are expressed as proportions.

## Results

Our study population consisted of 103 adults who tested positive on RT-PCR for COVID-19 infection and who underwent chest CT scans at our hospital. Of them, 97 had positive findings on chest CT scans and were included in the study. Six patients were excluded as they had no chest CT findings despite testing RT-PCR positive. In our study group, 75 patients were male and 22 patients were female. The highest percentage of patients in our study belonged to the 61-65 years age group followed by the 41-45 years age group. The percentage of each salient chest CT finding in the study group is represented in Table 1.

**Table 1:** Summary of all the features assessed on chest CT of study patients GGO: ground-glass opacities.

CT features	CT features	No. of patients	Percent
GGO		74	76.2
Consolidation		89	91.7
Crazy-paving		45	46.4
Vascular dilatation		75	77.3
Traction bronchiectasis		8	8.2
Subpleural bands		51	52.5
Architectural distortion		32	32.9
Parenchymal bands		36	37.1
Ancillary findings	Mediastinal lymphadenopathy	52	53.6
	Pleural effusion	2	2.06
	Pericardial effusion	39	40.2
	Pleural thickening	24	24.7
	CT halo sign	2	2
Underlying lung disease		6	6.18
Disease distribution	Unilateral	3	3
	Bilateral	94	96.9
Symmetry	Symmetric	25	25.7
	Asymmetric	78	80.4
Predominance of opacities on axial sections	Anterior	2	2.0
	Lateral	63	64.9
	Posterior	79	81.4
Distribution of opacities on axial sections: GGO, consolidation, or both	Central	0	0
	Peripheral	45	46.4
	Both	52	53.6
Lobes of lungs involved	Right upper lobe	92	94.8
	Right middle lobe	88	90.7
	Right lower lobe	93	95.8
	Left upper lobe	8	8.2
	Left lower lobe	96	98.9

From Table 1, we can infer that consolidation is the most common chest CT finding followed by vascular dilatation and ground-glass opacities. Subpleural bands were seen in 53% while a crazy-paving appearance was seen in 46% of patients. Linear and curvilinear parenchymal bands were seen in 37% of patients. Architectural distortion was seen in 33% of patients.

Among the ancillary features, mild mediastinal lymphadenopathy was the most common CT finding followed by minimal pericardial effusion. Minimal pleural thickening was seen in 25% while pleural effusion and CT halo signs were seen in about 2% of the patients each. In our study, five patients had underlying lung disease, two of them had paraseptal and centriacinar emphysema, two had old pulmonary tuberculosis, and one had calcified mediastinal tubercular lymphadenopathy.

Three of the study patients had a unilateral distribution of pulmonary opacities and 94 had bilateral opacities. Of all patients, 24% had near symmetric distribution while 76% had an asymmetric distribution of pulmonary opacities.

Regarding the distribution of consolidation, ground-glass opacities, and a combination of both on chest CT, 46% of patients had peripherally distributed opacities and 54% had both central and peripherally distributed opacities. Regarding the predominant location of consolidation, ground-glass opacities, and a combination of both on chest CT, 81% of patients had posterior predominance followed by lateral predominance. Of the study patients, 58% had combined lateral and posterior predominance of the above opacities, and 10% had nearly equal distribution along the anterior, lateral, and posterior aspects of the lung parenchyma. These findings are represented in Table 2.

**Table 2:** Predominant location of GGO and consolidation on axial CT sections in the study patients GGO: ground-glass opacities.

Predominance of opacities	Number of patients	Percentage of patients
Anterior	2	2
Lateral	63	64.9
Posterior	79	81.4
Combined lateral and posterior	56	57.7
Combined anterior and lateral	1	1
Combined anterior, lateral, and posterior	1	1
Equal distribution	10	10.3

Regarding lobes of the lung involved, the left lower lobe was involved most commonly, followed by the right lower lobe, right upper lobe, and right middle lobe in descending order.

As described earlier, all the study patients were classified into four groups: early, progressive, peak, and late stages. Table 3 shows the number and percentage of patients in each stage of the infection in our study.

**Table 3:** The number and percentage of patients in each stage of COVID-19 pneumonia in the study

Stage of COVID-19 pneumonia	Number of patients	Percentage of patients
Early stage	9	9.3
Progressive stage	45	46.4
Peak stage	39	40.2
Resorption stage	10	10.3

The two most important findings of COVID-19 pneumonia on the chest CT in this study were ground-glass opacities and consolidation. Table 4 shows the number and percentage of patients in each stage of COVID-19 pneumonia having consolidation and ground-glass opacities.

**Table 4:** The number and percentage of patients with ground-glass opacities and consolidation in each stage of COVID-19 pneumonia GGO: ground-glass opacities.

Stage of COVID-19 pneumonia	Total number of patients	Number of patients with GGO	Percentage of patients with GGO	Number of patients with consolidation	Percentage of patients with consolidation
Early stage	9	5	55.5%	0	0%
Progressive stage	45	37	82.2%	41	91.1%
Peak stage	39	29	74.3%	39	100%
Late stage	10	2	20%	9	90%

From Table 4, we can infer that the percentage of patients with ground glass opacities is about 55% during the early stage and increases to 82% during the progressive stage. It remains high at about 74.3% during the peak stage and declines to about 20% during the late stage of COVID-19 pneumonia. As regards consolidation, no patients in our study in the early phase of COVID-19 pneumonia had consolidation and about 91% had consolidation during the progressive stage. Of the patients, 100% had consolidation during the peak stage and 90% had consolidation during the late stage of the infection.

We can summarily infer from the above data that ground-glass opacities are an early feature of the infection on the chest CT. They persist throughout the acute stage and subside during the late stage of the infection, from 14 days onwards. On the other hand, consolidation begins to appear in the majority of patients after four days of the symptom onset and remains a dominant feature throughout the course of the infection. The rest of the chest CT findings are as given in Table 5.

**Table 5:** Percentage of patients with other CT features in each stage of COVID-19 pneumonia CP: crazy paving; VD: vascular dilatation; TB: traction bronchiectasis; SPB: subpleural bands; AD: architectural distortion; PB: parenchymal bands.

Stage of COVID-19 pneumonia	CP	VD	TB	SPB	AD	PB
Early stage	2	0	0	0	0	1
Progressive stage	24	29	1	22	14	13
Peak stage	17	35	5	23	14	16
Late stage	2	9	2	6	4	6

From Table 5, we conclude that a crazy-paving appearance on chest CT is present in a few patients during the early stage and peaks during the progressive stage. It persists during the peak stage and declines during the late stage of COVID-19 pneumonia. Vascular dilatation is also present in a significant percentage of patients during the progressive stage, peaks during the peak stage, and declines during the late stage of pneumonia. Traction bronchiectasis is not present in a significant percentage of patients during any stage of COVID-19 pneumonia and may only represent sequelae of disease resolution. Subpleural and parenchymal bands are present in the highest percentage during the progressive and peak stages. They decline during the late stage of COVID-19 pneumonia. The presence of architectural distortion mirrors the time trend exhibited by subpleural and parenchymal bands on chest CT in affected patients.

**Figure 1:**
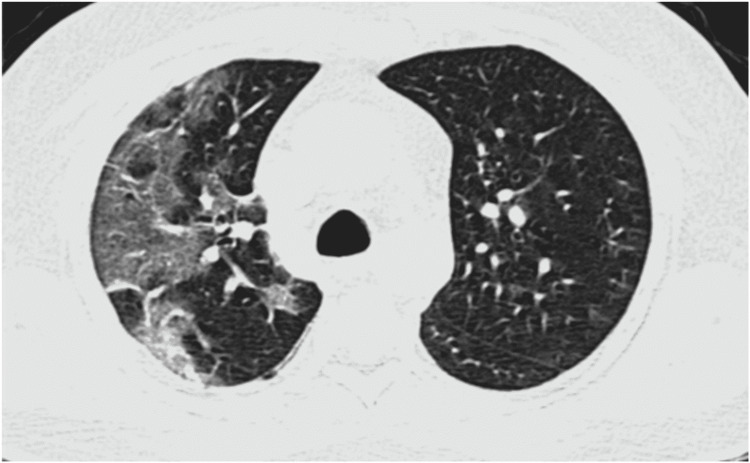
Axial CT section of the chest showing ground-glass opacities in a patient with COVID-19 pneumonia

**Figure 2:**
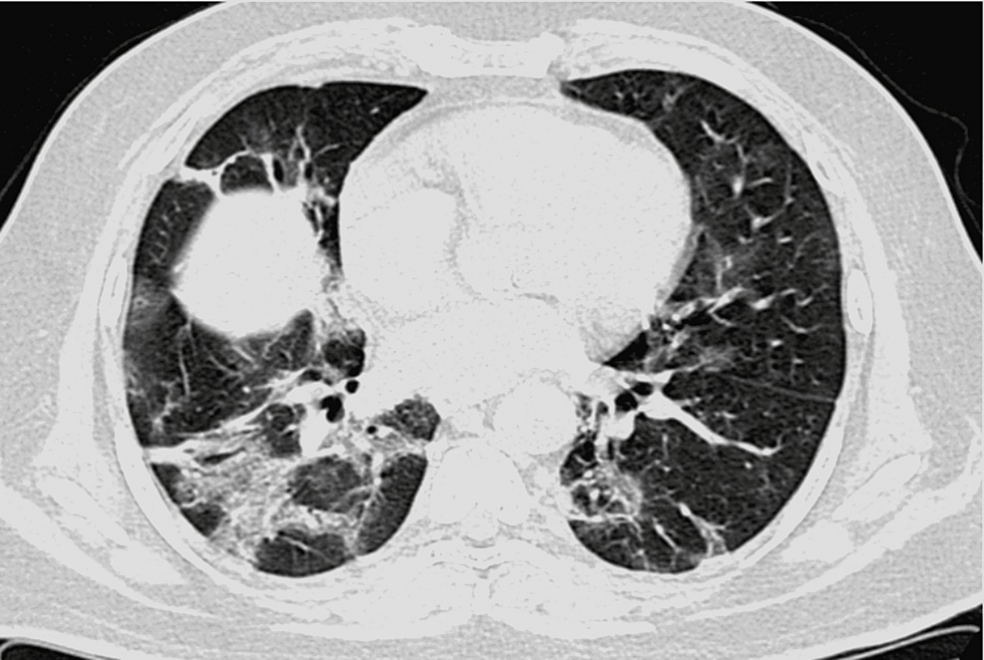
Axial CT section of the chest showing consolidation in a patient with COVID-19 pneumonia

**Figure 3:**
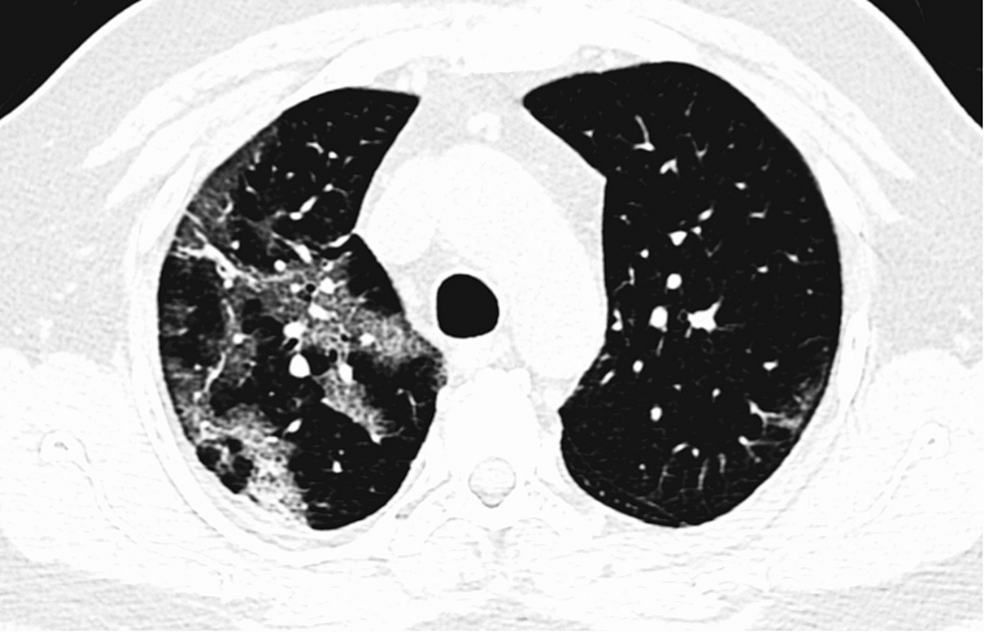
Axial CT section of the chest showing the “crazy-paving” appearance of ground-glass opacities with intralobular septal thickening in a patient with COVID-19 pneumonia

**Figure 4:**
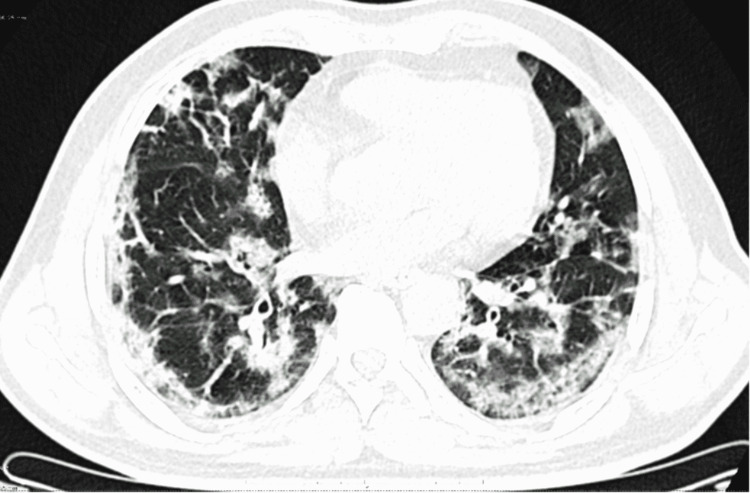
Axial CT section of the chest showing parenchymal bands and peripheral subpleural predominant distribution of opacities in a patient with COVID-19 pneumonia

The salient CT scan findings of COVID-19 pneumonia in our study were ground-glass opacities, consolidation, “crazy-paving” appearance (ground-glass opacities with intralobular septal thickening), minimal vascular dilatation, architectural distortion, traction bronchiectasis, and subpleural and linear parenchymal bands. Figures 1-4 are axial CT sections of the chest showing ground-glass opacities, consolidation, crazy-paving appearance, and parenchymal and subpleural bands in different patients with COVID-19 pneumonia in our study.

## Discussion

Among the various CT findings, ground-glass opacities, consolidation, and a combination of both were the most important features. These two features dominated the CT appearance of COVID-19 pneumonia in acute, progressive, and peak stages of the disease process. They also showed a distinctive pattern and trend over time during the course of the infection. The other findings of crazy-paving appearance, minimal vascular dilatation, architectural distortion, traction bronchiectasis, and subpleural and linear parenchymal bands were present in varying combinations depending upon the degree and stage of pneumonia at which the CT scan was performed.

The CT scan findings showed a distinct pattern of changes at different times during the course of the disease. Patients in the early stage showed predominant simple ground-glass opacities of mild to moderate degree. Patients in the progressive stage showed mild and moderate degrees of consolidation and ground-glass opacities with a crazy-paving appearance, minimal vascular dilatation, subpleural and parenchymal bands, and architectural distortion. Patients in the peak stage showed progression of these findings and during the late stage showed interval resolution of some findings. The distribution of the above-described opacities was asymmetric, with peripheral subpleural predominance involving the posterior, lateral, and both the above locations of the chest in varying proportions. The pulmonary opacities were associated with apicobasal gradient as evidenced by the preferential involvement of bilateral lower lobes in our study.

The findings in the present study are in broad agreement with the retrospective descriptive study by Bhandari et al. [1]. They investigated and quantified the severity of COVID-19 infection on HRCT and determined its relationship with clinical parameters. HRCT chest of their study population showed a variety of opacity characteristics; nearly half of whom showed typical ground-glass opacities while the other half showed a mixed pattern of ground-glass opacities and consolidation. CT scans performed in the early stage of pneumonia were characterized by ground-glass opacities and consolidation tended to dominate in the late stage. The distribution of opacities was mostly peripheral and posterior involving the lower lobes more frequently [1].

Kong et al. conducted a study to determine the chest CT manifestations of COVID-19 and its temporal evolution [11]. They concluded that the main imaging features of COVID-19 infection are simple ground-glass opacities with or without crazy-paving appearance and consolidation in the subpleural aspect of bilateral lungs. The lung lesions of mild COVID-19 pneumonia may improve significantly or disappear in a short period after treatment, with a good prognosis. Fibrosis-like stripes may be a sign of atelectasis of subsegment lung tissue in COVID-19 infection [11].

Pan et al. studied the time course of lung changes at chest CT during recovery from mild COVID-19 infection. They found that in patients recovering from mild COVID-19 infection, lung abnormalities on chest CT scans showed the greatest severity about 10 days after symptom onset [12].

Liang et al. studied the evolution of CT findings in patients with mild COVID-19 pneumonia and found that bilateral ground-glass opacities are the predominant features and CT findings changed during different time intervals three weeks after symptom onset [13].

Zhou et al. studied the evolution of chest CT findings from admission to follow-up in patients with COVID-19 pneumonia. They concluded that the dynamic evolution of CT features of moderate to severe COVID-19 pneumonia followed a specific pattern over time. During illness days zero to five, ground-glass opacity was the main pattern. On illness days 6-11, the main CT features were consolidation and reticular pattern. The consolidation and reticular pattern gradually diminished during illness days 12-23, and the reticular pattern and simple ground-glass opacities increased. When illness days were more than 24, the reticular pattern and simple ground-glass opacities gradually decreased until complete resolution. The highest CT score was on illness days 6-11 during which the lung abnormalities on chest CT were most severe [14]. The findings in our study are very similar to the findings of this study.

In an original research article by Kwee TC and Kwee RM, four stages of COVID-19 at chest CT have been described: (a) early stage (zero to five days after symptom onset) manifested by normal findings or ground-glass opacities; (b) progressive stage (five to eight days after symptom onset) characterized by increased ground-glass opacities and crazy-paving appearance; (c) peak stage (9-13 days after symptom onset) characterized by progressive consolidation; and (d) late stage (more than 14 days after symptom onset) characterized by a gradual decrease of consolidation and ground-glass opacities while signs of fibrosis may manifest. They noted that the temporal evolution and extent of lung abnormalities are heterogeneous among different patients, depending on disease severity [10].

There are important limitations of this study. First, ours was a retrospective, cross-sectional study that gives a “snap-shot” of the disease at one point in time in a patient. We did not carry out a longitudinal study except in a few patients in whom follow-up CT scans were performed dictated by clinical needs. Hence, the inferences drawn from the statistical analysis may not be an accurate reflection of the pathophysiologic sequence of events or the extent and severity of lung injury inflicted by the virus. Second, there was an inherent selection bias in that the majority of patients in the study were those who were clinically in the progressive and peak stages of the disease with fewer patients in the early and late stages of the disease. Third, many patients who were admitted to the ICU and diagnosed with severe or critical pneumonia did not undergo CT scan studies due to tachypnoea and the need for ventilatory support. Hence, these patients have not been studied. The inherent heterogeneity in patient inclusion and differences in disease extent, severity, stage, and duration in different patients may impact the CT features to some extent and will have a bearing on the final disease outcome. These factors are beyond the scope of this study.

## Conclusions

The RT-PCR test used in the primary diagnosis of COVID-19 infection is time-consuming, requires the ready availability of an adequate number of test kits, and has a certain proportion of false-negative results if the viral load is insufficient. CT scans require much less time, can be easily performed with minimal patient discomfort, and results are available within a short span of time. Therefore, it is an essential aid in the non-invasive detection of moderate and severely infected patients with COVID-19 pneumonia. We can summarize that in the current COVID-19 pandemic, in patients who present with clinical features meeting the WHO criteria or case definition of COVID-19 infection, the chest CT features of ground-glass opacities and consolidation in varying combination with a characteristic asymmetric peripheral subpleural distribution in the posterior and lateral aspects of the lung are virtually pathognomonic of COVID-19 pneumonia. These CT features permit rapid non-invasive detection of the extent and severity of COVID-19 pneumonia enabling appropriate treatment to be instituted at the earliest and are thus life-saving in resource-constrained environments. However, it must be noted that chest CT scans are not a substitute for the primary diagnosis of COVID-19 infection, which relies on the demonstration of the presence of the offending virus in the respiratory tract.
